# Parenting Sense of Competence and Maternal Employment Guilt Among Female Healthcare Professionals with Young Children in Türkiye: A Cross-Sectional Study

**DOI:** 10.3390/healthcare14213568

**Published:** 2026-10-22

**Authors:** Merve Akça, Emine Güdek Seferoğlu

**Affiliations:** 1 Institute of Postgraduate Education, Kütahya Health Sciences University, Kütahya 43020, Türkiye; merveakca262@gmail.com; 2 Faculty of Health Sciences, Kütahya Health Sciences University, Kütahya 43020, Türkiye

**Keywords:** healthcare professionals, nurses, midwives, parenting sense of competence, maternal employment guilt, work–family balance

## Abstract

Background: Female healthcare professionals with young children may experience maternal employment guilt when they perceive that paid work limits the time and attention they can give their children. This study examined the relationship between parenting sense of competence and maternal employment guilt among nurses and midwives with children aged 0–6 years. It also explored associated sociodemographic and work-related factors during the COVID-19 post-lockdown transition. Methods: This cross-sectional study was conducted between May and August 2021 in hospital and primary healthcare settings in western Türkiye. Of 174 eligible healthcare professionals, 150 participated. Data were collected using the Parenting Sense of Competence Scale–Mother Form (PSOC), the Maternal Employment Guilt Scale (MEGS), and a personal information form. Group comparisons, Spearman correlations, and multivariable linear regression were performed. Results: The mean total PSOC score was 55.89 ± 7.47, and the mean MEGS score was 62.27 ± 15.72. Parental satisfaction showed the largest observed inverse correlation with maternal employment guilt among the PSOC scores examined (ρ = −0.324; *p* < 0.001). The correlation with total PSOC was smaller (ρ = −0.177; *p* = 0.030), and total PSOC was not statistically significant in the adjusted linear model (*p* = 0.071). The overall adjusted linear model was not statistically significant (*p* = 0.089). In supplementary exploratory models, satisfaction remained inversely associated with guilt after adjustment (Holm-adjusted *p* = 0.015), although its overall model test was nonsignificant (*p* = 0.081). Day-only work and a 40-h working week were associated with higher scores on some PSOC measures in exploratory comparisons. Conclusions: Among nurses and midwives with young children, maternal employment guilt was inversely associated with satisfaction in the parenting role. Work schedule and weekly working hours were also associated with some parenting sense of competence scores. These cross-sectional findings highlight specific aspects of healthcare professionals’ work–family experience for further investigation.

## 1. Background

The participation of women in employment is a key determinant of social welfare and economic development. Despite marginal gains over the past two decades, women’s labor force participation remains substantially lower than that of men, with unequal childcare responsibilities contributing to this gap. According to the International Labour Organization, in 2023 the global labor force participation rate among adults aged 25–54 was 64.5% for women compared with 92% for men [1]. Among women in this age group, those with children under six had labor force participation rates 12 and 14 percentage points lower in couple and extended-family households, respectively, than those without young children [1]. In Türkiye, this disparity is also pronounced: in 2022, the employment rate among women aged 25–49 with children under the age of three was 28.0%, compared with 56.2% among women in the same age group without children under three [2].

Nursing and midwifery are female-dominated professions characterized by demanding workloads, shift-based schedules, continuous patient care, and substantial emotional responsibilities. Nurses frequently report difficulty balancing work and family roles, which has been associated with burnout, anxiety, feelings of inadequacy, and parenting-related guilt [3,4]. Evidence from Türkiye indicates that shift work can restrict the social and family lives of nurses and midwives and reduce the time available for family responsibilities [5]. A systematic review similarly found that nurse-parents experience dual-role conflict, compromised self-care, and career trade-offs [6]. Including both professions in the present study reflects their shared work–family demands without assuming that their occupational experiences are identical.

Previous research has explored maternal guilt among nurses in Türkiye and the associations of parenting self-efficacy with parental well-being and social support [4,7,8]. In another population, Maclean et al. found that higher parenting self-efficacy was associated with lower work-interfering-with-family guilt among working or studying mothers with children aged under 13 years [9]. Their study assessed self-efficacy using the Parental Self-Agency Measure and guilt using the Work-Interfering-with-Family Guilt Scale; it did not examine the efficacy, interest, and satisfaction dimensions of parenting sense of competence together. Thus, an association between parenting self-efficacy and employment-related guilt has been investigated, but this evidence does not establish whether guilt is similarly related to the distinct dimensions of parenting sense of competence among nurses and midwives with young children. The present study extends this evidence by examining parental efficacy, interest, and satisfaction alongside total parenting sense of competence in relation to maternal employment guilt. Its contribution lies in combining this multidimensional assessment with the work–family context of nurses and midwives raising children aged 0–6 years in Türkiye. The adjusted analysis further examines the association between total parenting sense of competence and maternal employment guilt while accounting for selected sociodemographic and work-related characteristics.

Maternal employment guilt refers to guilt experienced when working mothers perceive that paid employment limits the time, attention, or emotional availability they can provide to their children. Such guilt may be particularly salient among mothers of young children, who require greater care and supervision [10]. The construct specifically concerns the perceived consequences of employment for the maternal role, rather than guilt about parenting more generally. Work–family conflict and employment-related guilt have been associated with parental stress, lower parental satisfaction and well-being, and intentions to leave paid employment [11,12,13]. Mothers may also restrict personal time or modify their parenting behavior in response to guilt [14].

Parenting sense of competence encompasses parents’ perceived efficacy, interest, and satisfaction in the parenting role [15,16]. These dimensions capture perceived capability in meeting parenting demands (efficacy), interest in the parenting role (interest), and satisfaction with the parenting experience (satisfaction). Previous research has linked parenting self-efficacy with parent and child well-being [7] and perceived social support [8]. Findings concerning self-efficacy alone, however, do not establish whether maternal employment guilt is similarly associated with all dimensions of parenting sense of competence. Examining these dimensions separately therefore allows a more differentiated assessment of the relationship.

Motherhood experiences are shaped by cultural and gender expectations. The ideology of intensive motherhood portrays the “good mother” as continuously available, emotionally invested, and primarily responsible for caregiving, often at the expense of her own needs [17,18]. Expectations that position motherhood as women’s primary responsibility may increase pressure on working mothers and intensify perceived conflict between employment and childcare [11,19]. When mothers perceive that employment prevents them from meeting these expectations, the discrepancy may be accompanied by employment-related guilt and less positive appraisals of their own parenting. This framework provides a rationale for examining the association between maternal employment guilt and parenting sense of competence. Examining efficacy, interest, and satisfaction separately allows exploration of how this association is expressed across different aspects of the parenting role. Intensive motherhood beliefs and gender-role expectations were not directly measured and are used here to guide the research question rather than as tested explanatory mechanisms.

Data were collected between May and August 2021, during the transition following Türkiye’s nationwide COVID-19 lockdown of 29 April–17 May 2021. Research from the pandemic period described stress and challenges to family life among healthcare-professional parents [20,21,22]. Individual pandemic-related exposures, including workload changes, childcare disruption, and infection concerns, were not measured in this study. COVID-19 therefore represents the context of data collection rather than a tested determinant of the observed associations.

This cross-sectional study examined the association between parenting sense of competence and maternal employment guilt among nurses and midwives with children aged 0–6 years in Türkiye. Specifically, it examined the associations of efficacy, interest, satisfaction, and total Parenting Sense of Competence Scale (PSOC) scores with Maternal Employment Guilt Scale (MEGS) scores; explored sociodemographic and work-related differences in these scores; and assessed the total PSOC–MEGS association after adjustment for selected sociodemographic and work-related characteristics. Professional title was not recorded as a separate variable; therefore, the sample’s composition by profession could not be reported and profession-specific differences could not be examined, limiting the generalizability of the findings to either profession separately.

## 2. Methods

### 2.1. Study Design and Population

This cross-sectional correlational study was conducted between May and August 2021 among female nurses and midwives working in a training and research hospital and family health centers in a city in western Türkiye. Eligibility criteria were having at least one child aged 0–6 years, living with the child, and being actively employed during the study period. Participation was voluntary and required written informed consent.

At the hospital, the researcher obtained information about the units employing nurses and midwives from the Director of Healthcare Services. The researcher then visited these units and consulted unit supervisors to identify employees who met the eligibility criteria. For primary healthcare settings, the researcher contacted the supervisors of all 20 family health centers in the city center by telephone to identify eligible employees. Not all centers necessarily had eligible employees. Participants from family health centers were classified within ambulatory units for analysis.

Through this process, 174 eligible healthcare professionals were identified. The study sought to include the entire accessible population rather than select a sample. All 174 were invited to participate; 24 declined, and 150 provided written informed consent and completed the questionnaires, yielding a participation rate of 86.2%. No formal sample-size calculation was performed before recruitment; the final sample size was determined by participation within the accessible population. The regression analyses were secondary and exploratory. The sample size was not planned to provide a specified power or precision for these multivariable models.

Nurses and midwives were considered together because the study focused on their shared work–family demands, without assuming identical occupational experiences. Professional title was not recorded separately, preventing the reporting of the sample’s distribution by profession and the examination of differences between the two groups.

All 150 questionnaires contained complete PSOC and MEGS responses. Three participants who reported using unpaid maternity leave did not specify its duration; analyses of leave duration therefore included only participants with available data.

### 2.2. Data Collection

Data were collected between May and August 2021 using paper-and-pencil questionnaires during the gradual normalization period following the nationwide COVID-19 lockdown. Eligible employees were identified and invited as described in Section 2.1. The researcher explained the study’s purpose, scope, and voluntary nature before participation.

Data collection took place outside treatment and care hours, in unit offices or staff break rooms. Healthcare professionals who agreed to participate were given a questionnaire enclosed in an unmarked envelope to be completed individually. Participants returned the completed questionnaires in sealed envelopes to the researcher or the responsible unit manager.

Throughout the data collection process, all COVID-19 precautionary measures, including mask use, physical distancing, and hand hygiene, were strictly observed.

### 2.3. Instruments Used in the Study

#### 2.3.1. Personal Information Form

A researcher-developed 16-item personal information form collected sociodemographic and work-related data based on the literature [3,4,5,21]. Age was recorded as 20–25, 26–30, 31–35, and >35 years, and weekly working hours as 40, 45, and ≥46 h; exact continuous values were unavailable. Age categories were combined into ≤30 and ≥31 years to avoid retaining the youngest category of only six participants and to provide a parsimonious age adjustment. This boundary followed the questionnaire categories rather than a validated clinical threshold. The working-hour response options were informed by the general weekly working-time provisions in Türkiye: 40 h for public servants under Article 99 of Law No. 657 [23] and a maximum normal working week of 45 h for employees covered by Article 63 of Labour Law No. 4857 [24]. For analysis, the 45-h and ≥46-h responses were combined to compare the general 40-h public-sector working week with reported working hours of ≥45. Participants were not classified by legal employment status. Work schedule was grouped as day-only or shift work, with the latter combining alternating day/night work and on-call duty. Work unit was classified as ambulatory, operating room/emergency, inpatient, or intensive care. Other characteristics included family type, income, personal and child health problems, childcare support, work experience, number and ages of children, and leave use and duration. The maternity leave questions referred to unpaid maternity leave; only use (yes/no), not duration, was included in the regression models.

#### 2.3.2. The Parenting Sense of Competence (PSOC)-Mother Form

Parenting sense of competence was measured using the Parenting Sense of Competence Scale–Mother Form (PSOC), developed by Gibaud-Wallston and Wandersman (1978) [25] and adapted into Turkish by Seçer et al. (2008) [16]. The 16-item, 5-point Likert scale comprises parental efficacy (7 items; range 7–35), parental interest (2 items; range 2–10), and parental satisfaction (7 items; range 7–35). Raw responses ranged from 1 (strongly agree) to 5 (strongly disagree). Positively worded items (1, 2, 4, 8, 10, 11, and 13) were recoded as 6 minus the raw response, while negatively worded items (3, 5, 6, 7, 9, 12, 14, 15, and 16) retained their raw codes, so that higher subscale and total scores indicated greater perceived parenting competence. Item-level recoding and scale-score calculations were rechecked against the raw responses during revision; this verification did not change the reported scores. The total score ranges from 16 to 80 and has no validated cut-off point. In the present sample, Cronbach’s alpha was 0.69 for efficacy, 0.82 for interest, 0.74 for satisfaction, and 0.67 for the total scale.

#### 2.3.3. Maternal Employment Guilt Scale

Maternal employment guilt was measured using the 15-item Maternal Employment Guilt Scale (MEGS), developed and validated in Turkish by Yüce Selvi and Kantaş (2019) [26]. Items are rated from 1 (does not describe me at all) to 6 (completely describes me). Total scores range from 15 to 90, with higher scores indicating greater maternal employment guilt. The scale has no validated cut-off point; therefore, scores were reported descriptively rather than categorized. Cronbach’s alpha was 0.95 in the present sample.

### 2.4. Data Analysis

Statistical analyses were performed using IBM SPSS Statistics 24 and independently verified from the raw response data. Frequencies, percentages, means, standard deviations, medians, and ranges were calculated. Distributional assumptions were evaluated using the Shapiro–Wilk test, skewness and kurtosis, histograms, and Q–Q plots; homogeneity of variance was assessed with Levene’s test. Efficacy and interest scores were compared using Mann–Whitney U or Kruskal–Wallis tests; means and standard deviations in the tables are descriptive for these rank-based analyses. Satisfaction, total PSOC, and MEGS scores were compared using independent-samples t tests or one-way ANOVA, with Welch’s tests for unequal variances. Distributional judgments considered graphical patterns, skewness, and group sizes alongside Shapiro–Wilk results, rather than normality-test *p* values alone. During revision, variance diagnostics were rechecked using median-centered Levene tests because of departures from normality. Tukey–Kramer-adjusted pairwise comparisons followed significant one-way ANOVA tests, and Dunn comparisons with tie correction and Bonferroni adjustment followed significant Kruskal–Wallis tests. Pairwise adjusted *p* values are reported in Table S4. Associations between scale scores were examined using Spearman’s rank correlation with bootstrap 95% confidence intervals based on 10,000 resamples.

MEGS total score was the dependent variable, and total PSOC score was the main independent variable, entered continuously. Total PSOC was selected to examine overall parenting sense of competence in relation to maternal employment guilt, consistent with the study objective and the total-score approach used in the Turkish adaptation. Covariates represented sociodemographic characteristics (age and education), caregiving needs and resources (child health status and childcare support), and employment conditions (work schedule, weekly working hours, unpaid maternity leave use, and work unit). The rationale for inclusion drew on literature concerning work–family conflict, parenting self-efficacy, and working-parent experiences [3,6,8,11,27]; selection was not restricted to significant bivariate associations. Work unit served as a contextual indicator of the clinical setting. All covariates were entered simultaneously. Binary covariates were coded 0 for reference categories and 1 for comparison categories; work unit was represented by three dummy variables. Reference categories were age ≤ 30 years, high school education, no child health problem, no childcare support, day-only work, a 40-h working week, no unpaid maternity leave use, and ambulatory units. The supplementary dimension-specific models used the same covariates and coding. These models were exploratory and did not establish a causal direction.

Heteroskedasticity-consistent HC3 standard errors were used for coefficient inference. Two-sided coefficient *p* values were based on z tests using the standard normal distribution, and 95% confidence intervals were calculated as B ± 1.96 × SE(HC3). Overall model significance was assessed using an HC3-robust Wald F test with 11 numerator and 138 denominator degrees of freedom in the total-score model (N = 150). Multicollinearity was assessed using variance inflation factors. All tests were two-sided, and *p* < 0.05 was considered statistically significant. Tukey and Bonferroni adjustments controlled pairwise comparisons within individual analyses, not multiplicity across the full set of bivariate tests. A single correction across all bivariate tests was not applied; overall group comparisons are reported with nominal p values and interpreted as exploratory. Isolated significant findings, particularly those close to p = 0.05, were treated as hypothesis-generating rather than confirmatory. The Holm adjustment for the three supplementary dimension-specific regression coefficients constituted a separate test family and did not adjust these bivariate comparisons. 

During revision, additional regression diagnostics were computed from the analysis data using Python 3.12.14, NumPy 2.3.5, and SciPy 1.17.0. Residual-versus-fitted, normal Q–Q, and partial-regression plots were examined. A centered quadratic PSOC term was added to the covariate-adjusted model to assess departure from linearity, with HC3 inference. Residual heteroskedasticity was assessed using the studentized Breusch–Pagan (Koenker) test. Influence screening used externally studentized residuals (absolute value > 3), leverage (>2k/n), and Cook’s distance (>4/n), where k includes the intercept. These were screening thresholds, not automatic exclusion criteria. All participants were retained in the main analysis; leave-one-out refits and a refit excluding all flagged observations assessed sensitivity. The quadratic model was an additional post hoc exploratory analysis (Table S3; Figure S1).

Three supplementary exploratory linear regression models replaced total PSOC with efficacy, interest, or satisfaction separately while retaining the same covariates and category coding as the total-score model. MEGS remained the dependent variable. Coefficient confidence intervals and *p* values used HC3-robust standard errors with a normal approximation; overall model tests used HC3-robust Wald F tests. Holm adjustment was applied across the three dimension-specific coefficient tests. These supplementary analyses were not prespecified.

Following peer review, supplementary exploratory comparisons assessed differences between the Spearman correlation of MEGS with satisfaction and its correlations with efficacy, interest, and total PSOC. Paired participant bootstrap resampling (50,000 resamples) preserved the dependence between scores; ranks were recalculated within each resample. Signed correlation differences were reported with percentile 95% confidence intervals and approximate two-sided centered-bootstrap *p* values. Holm correction was applied separately across these three comparisons (Table S2). The total-score comparison was secondary because total PSOC includes satisfaction items. These analyses were not prespecified. Satisfaction was selected for these comparisons after inspection of the observed correlations; Holm adjustment does not account for this selection.

### 2.5. Ethical Consideration

Before starting the study, approval was obtained from a university Ethics Committee (No. 2021/01-19) and permission was received from the institutions where the study was conducted. Permission was obtained from the authors who conducted the validity and reliability studies of the scales. After explaining the purpose of the study, written consent was obtained from those who voluntarily agreed to participate.

## 3. Results

### 3.1. Participant Characteristics

Table 1 presents participant characteristics. Of the participants, 57.3% were aged ≥ 31 years, 87.3% were university graduates, 97.3% were married, 91.3% lived in a nuclear family, and 50.0% reported income equal to expenses. Nearly half had one child (49.3%), and 44.0% had a youngest child aged 3–4 years. Most participants reported no personal health problem (78.0%), no health problem in their child (90.7%), and access to childcare support (88.0%).

Overall, 44.0% had 6–10 years of work experience, 36.0% worked in intensive care, 70.0% worked shifts, and 58.0% worked at least 45 h per week. A total of 107 participants (71.3%) reported using unpaid maternity leave. Among the 104 participants who reported its duration, 68 (65.4%) reported one year and 36 (34.6%) reported two years; duration was missing for three participants.

### 3.2. Findings on the PSOC, Its Sub-Dimensions, and Related Factors

Distribution checks showed pronounced departures from normality for efficacy and interest, supporting rank-based comparisons. Satisfaction and total PSOC were more symmetric, although some within-group Shapiro–Wilk tests were significant; MEGS showed negative skew. Mean-based comparisons were retained as exploratory assessments of score means. Median-centered Levene tests identified unequal MEGS variances by education (*p* = 0.002) and weekly working hours (*p* = 0.045), among other comparisons, supporting the use of Welch’s tests. Diagnostic tests were less informative in very small groups, particularly the poor-income category (*n* = 4); comparisons involving such groups should therefore be interpreted cautiously.

The mean scores were 27.68 ± 3.87 for parental efficacy, 6.85 ± 2.43 for parental interest, 21.36 ± 5.53 for parental satisfaction, and 55.89 ± 7.47 for total PSOC (Table 2). Because the PSOC has no validated cut-off point, these scores were not categorized as low, moderate, or high.

Exploratory comparisons indicated higher parental interest, satisfaction, and total PSOC scores among day-only workers and those working 40 h per week than among shift workers and those working ≥ 45 h, respectively. Other sociodemographic and work-related comparisons are presented in Table 3. Overall group-test *p* values were not adjusted across the full set of comparisons; these findings are hypothesis-generating.

### 3.3. Findings on the MEGS and Related Factors

The mean MEGS score was 62.27 ± 15.72 (possible range 15–90). Its position above the theoretical midpoint of 52.5 is a descriptive comparison only, as no validated cut-off is available. Nominal comparisons indicated higher scores among high-school graduates than university graduates (*p* = 0.048) and among mothers whose children did not have a health problem (*p* = 0.003). These findings are exploratory; the education comparison is particularly tentative given the number of tests. Detailed comparisons are reported in Table 3.

### 3.4. Findings on the Relationship Between PSOC and MEGS

Maternal employment guilt was weakly and inversely correlated with parental satisfaction (ρ = −0.324, 95% CI −0.466 to −0.162; *p* < 0.001) and total PSOC (ρ = −0.177, 95% CI −0.336 to −0.013; *p* = 0.030) (Table 4). Efficacy and interest were not significantly correlated with MEGS. The adjusted regression model was not statistically significant overall (R^2^ = 0.124, adjusted R^2^ = 0.054; HC3-robust Wald F(11,138) = 1.659; *p* = 0.089). Total PSOC was not independently associated with MEGS (B = −0.456, 95% CI −0.950 to 0.038; *p* = 0.071). The coefficient for having a child with a health problem was −12.77 (95% CI −23.30 to −2.23; *p* = 0.018), but this exploratory coefficient warrants caution given the nonsignificant overall model and the small subgroup (*n* = 14). All variance inflation factors were below 3 (Table 5).

Diagnostics identified heteroskedasticity (Koenker test, *p* = 0.007) and evidence of departure from linearity: the added quadratic PSOC term was negative (B = −0.063, 95% CI −0.101 to −0.025; HC3 *p* = 0.001). The exploratory quadratic model had R^2^ = 0.195 and HC3-robust Wald F(12,137) = 2.331 (*p* = 0.010). Residual Q–Q inspection showed tail deviations, although the Shapiro–Wilk test was nonsignificant (*p* = 0.146). Twelve observations exceeded Cook’s distance 4/n; one exceeded leverage 2k/n, and one had an externally studentized residual below −3. Thirteen observations met at least one screening criterion. Excluding these observations in a sensitivity refit yielded a linear PSOC coefficient of −0.347 (95% CI −0.758 to 0.064; *p* = 0.098). Across leave-one-out refits, the linear coefficient remained negative (−0.543 to −0.358), but *p* values ranged from 0.026 to 0.135. The quadratic term remained negative in all leave-one-out refits and in the flagged-case sensitivity refit (*p* = 0.007). These findings indicate sensitivity of linear-model inference and possible nonlinearity rather than absence of an adjusted relationship (Table S3; Figure S1).

Supplementary dimension-specific regressions are presented in Table S1. Satisfaction was inversely associated with MEGS after covariate adjustment (B = −0.916, 95% CI −1.555 to −0.277; *p* = 0.005; Holm-adjusted *p* = 0.015). Efficacy and interest were not statistically significant (*p* = 0.822 and *p* = 0.685, respectively). The overall satisfaction model was not statistically significant (HC3-robust Wald F(11,138) = 1.694; *p* = 0.081; R^2^ = 0.161). These results are exploratory.

In supplementary paired-bootstrap comparisons, the satisfaction–MEGS correlation was more negative than the efficacy–MEGS and interest–MEGS correlations (Holm-adjusted *p* = 0.0025 for each); its difference from the total PSOC–MEGS correlation was also significant (Holm-adjusted *p* = 0.0087), with the latter constituting a secondary part–whole comparison (Table S2).

## 4. Discussion

Parental satisfaction showed the largest observed inverse correlation with MEGS among the PSOC scores examined. Supplementary paired-bootstrap comparisons indicated a more negative correlation for satisfaction than for efficacy and interest after Holm correction (Table S2). These post-review analyses compare signed correlations; they do not establish that satisfaction has a uniquely causal role or that the association is independent of other parenting dimensions. The discussion considers this exploratory pattern in relation to previous research, followed by the secondary group comparisons and the measurement properties of PSOC.

### 4.1. Parenting Satisfaction and Maternal Employment Guilt

Öke Karakaya et al.’s qualitative study of nurses in Türkiye identified feelings of inadequacy and the tension between career success and being a good mother [4]. These accounts provide an occupational and cultural context for the present association between employment guilt and lower parenting satisfaction. However, qualitative accounts of inadequacy are not equivalent to PSOC efficacy scores: they cannot establish an association between the measured constructs. Our findings add a quantitative distinction between satisfaction with parenting and perceived efficacy, rather than treating all negative parenting evaluations as interchangeable.

Aarntzen et al. found that work–family guilt was associated with lower happiness and lower satisfaction with combining work and family in a daily diary study [13]. The inverse association with PSOC satisfaction observed here is directionally consistent with that finding, but the outcomes differ: satisfaction with the work–family combination is not the same as satisfaction in the parenting role. Their repeated measurements also captured daily fluctuations, whereas the present study examined differences between participants at one time point. The studies therefore provide complementary evidence rather than a direct replication.

Maclean et al., in a survey of 1375 working or studying mothers of children aged ≤ 12 years, found that higher parenting self-efficacy was associated with less work-interfering-with-family guilt [9]. PSOC efficacy was not significantly associated with MEGS in the present sample. However, a significant association in one study and a nonsignificant association in another do not, by themselves, establish a difference between studies. Differences in populations, child age ranges, measurement instruments, and reliability limit direct comparison. Examining the PSOC dimensions separately identifies an association with parenting satisfaction that a focus on self-efficacy alone would not capture. The supplementary comparisons support a difference between the signed correlations in this sample, but do not establish a unique explanatory contribution of satisfaction. Differences in measurement reliability and the exploratory nature of the analyses remain relevant to interpretation.

The inverse bivariate association between total PSOC and MEGS (*p* = 0.030) was not statistically significant in the covariate-adjusted linear model (*p* = 0.071), and the overall linear model was also nonsignificant (*p* = 0.089). The bivariate finding therefore does not establish an association independent of the included covariates; the adjusted result also does not rule out an association. Sociodemographic and work-related group differences likewise remain exploratory observations rather than established explanatory factors. In supplementary exploratory models, the satisfaction coefficient remained significant after covariate adjustment and Holm correction across the three dimension-specific tests, whereas efficacy and interest were not significant (Table S1). However, the overall satisfaction model was nonsignificant. This coefficient warrants further investigation, but neither the supplementary analysis nor the unadjusted comparisons provide confirmatory evidence of independent determinants of maternal employment guilt.

The additional diagnostic analysis identified a negative quadratic PSOC term, suggesting that a single linear coefficient may not adequately summarize the adjusted association. The linear coefficient also showed sensitivity to individual observations. The quadratic finding was obtained during revision and remains exploratory; it does not establish a validated nonlinear pattern or a clinically meaningful threshold. Together with the modest reliability of total PSOC, these findings limit interpretation of the linear model’s nonsignificant result as evidence against an association.

The intensive motherhood framework introduced earlier offers one possible interpretation of the inverse association between maternal employment guilt and parenting satisfaction. Expectations that a “good mother” should remain continuously available may create a perceived discrepancy between employment demands and idealized caregiving standards [17,18]. Research in Türkiye has linked maternal employment guilt with internalized sexism [28]. Qualitative research from Iceland and a review of maternal guilt also describe expectations of constant maternal availability and responsibility as a context for guilt [29,30]. Such a discrepancy could be relevant to how mothers experience satisfaction in the parenting role, without necessarily implying lower perceived parenting capability. The distinction between satisfaction and efficacy observed here makes this a useful question for further research. However, intensive motherhood beliefs, gender-role expectations, and perceived discrepancies were not measured; the findings therefore do not test this framework or establish it as the mechanism underlying the association. The cross-sectional design also cannot determine whether employment guilt precedes reduced parenting satisfaction or vice versa.

### 4.2. Sociodemographic and Work-Related Findings

The group comparisons provide contextual findings rather than explanations of the main association. Differences by work schedule and weekly hours are relevant to literature on work–family balance among nurses and midwives [5,31], but do not demonstrate that changing schedules would improve parenting experiences. Likewise, the nominal education–MEGS difference (*p* = 0.048) is tentative given the number of comparisons and was not supported in the adjusted model. The MEGS mean is descriptive: neither its position above the theoretical midpoint nor comparisons between groups establish a clinically significant level of guilt. Other Turkish studies using MEGS have examined psychological resilience and workplace breastfeeding support [32,33], illustrating aspects of employment guilt beyond the parenting competence scores assessed here.

Higher parental satisfaction among participants without childcare support and lower employment guilt among mothers of children with health problems require cautious interpretation. Support was recorded only as present or absent; 18 participants reported no support. Child health problems were reported by 14 participants, without clinical details. Residual confounding by unmeasured factors, such as support quality, family relationships, or the severity of the child’s condition, may contribute to these patterns. Reverse associations are also possible: mothers experiencing greater parenting difficulties may be more likely to seek childcare support, rather than support itself reducing satisfaction. These explanations were not tested. Small subgroups and multiple exploratory comparisons also leave open the possibility of chance findings. Although the child-health coefficient was significant in the adjusted model, the overall model was nonsignificant. These findings therefore do not establish a benefit of lacking support or an independent predictive role for child health status. Other demographic and occupational comparisons are presented in Table 3 without individual explanatory claims.

### 4.3. Measurement Considerations

The measurement properties of PSOC warrant caution. Internal consistency was modest for the total score (α = 0.67) and efficacy dimension (α = 0.69), compared with interest (α = 0.82) and satisfaction (α = 0.74). The total score was retained to address the study objective concerning overall parenting sense of competence and in accordance with the total-score approach used in the Turkish adaptation. However, this scoring convention does not, by itself, establish the psychometric adequacy of the total score in the present sample. Limited internal consistency may introduce measurement imprecision and affect the estimated associations. The total-score regression is therefore interpreted as secondary and exploratory. Dimension-specific results are reported separately, including supplementary adjusted analyses (Table S1); these provide additional information but do not resolve the total score’s measurement limitations. All items were retained.

## 5. Strengths and Limitations

This study addressed an underexplored population—female nurses and midwives with children aged 0–6 years—during the COVID-19 post-lockdown transition and reached 86.2% of the accessible population. The use of previously adapted instruments, complete scale responses, and verification of scoring from the raw responses supported the analysis, although internal consistency was limited for some PSOC scores.

Several limitations should be acknowledged. The cross-sectional design precludes causal inference, and recruitment from healthcare settings in one city limits generalizability. Pandemic-specific exposures, such as COVID-unit assignment, infection concerns, childcare closure, and family separation, were not measured; COVID-19 should therefore be interpreted as context rather than a tested cause. Professional title was not recorded separately, preventing reporting of the nurse–midwife composition and profession-specific analyses; representativeness for either profession cannot be assessed. Although participation was high (86.2%), 24 eligible healthcare professionals declined participation. Their parenting experiences and maternal employment guilt may have differed from those of participants; therefore, non-response bias cannot be ruled out. Child health conditions were not characterized by type or severity, and childcare support was recorded only as present or absent. Three participants did not report unpaid maternity leave duration. The modest internal consistency of total PSOC and efficacy limits confidence in associations involving these scores. Measurement imprecision may attenuate associations; nonsignificant findings should not be interpreted as establishing the absence of a relationship. The supplementary dimension-specific models were undertaken during revision and remain exploratory. All outcomes were self-reported. No correction was applied across the full set of bivariate tests, leaving an increased risk of false-positive findings despite adjustments within individual post hoc comparisons. Age and weekly working hours were collected using predefined questionnaire categories rather than exact continuous values. Combining age categories into ≤30 and ≥31 years and working-hour categories into 40 and ≥45 h reduced information and may have obscured differences within these broader groups. These analytical groupings should not be interpreted as validated thresholds. The questionnaire did not separately capture 41–44 weekly hours. Some group distributions departed from normality, and diagnostic tests could not reliably establish distributional adequacy in very small subgroups, especially the poor-income category (*n* = 4). Mean-based comparisons involving these groups are particularly tentative.

The available sample size was not determined specifically for multivariable regression. Each model estimated 11 predictor coefficients, excluding the intercept, from 150 participants. This sample size, together with small numbers in some covariate categories, may limit the precision and stability of adjusted estimates. A sample-size calculation for a bivariate correlation would not establish adequacy for these models. Although HC3-robust standard errors and 95% confidence intervals were reported, this approach does not compensate for limited sample information or sparse categories. Adjusted findings should therefore be interpreted as exploratory; nonsignificant results do not exclude potentially relevant associations. Regression diagnostics also indicated possible nonlinearity and sensitivity of linear-coefficient inference to individual observations. The quadratic analysis was post hoc and requires independent replication; HC3 standard errors address heteroskedasticity but do not correct functional-form misspecification.

## 6. Recommendations for Future Research

Longitudinal and multicenter studies could test whether the association with parenting satisfaction persists across healthcare settings and changes over time. Recording professional title would permit separate analyses for nurses and midwives. More detailed measures of child health, childcare arrangements, managerial support, and work schedules could help explain the group differences observed here. Interviews may be useful for understanding how healthcare professional mothers describe employment guilt in their own words.

## 7. Implications for Policy and Practice

The findings identify questions for policy and practice research rather than demonstrating the effectiveness of organizational changes. Given the exploratory, cross-sectional analyses and nonsignificant overall adjusted linear model, differences by work schedule and weekly hours do not establish that altering these conditions would improve parenting satisfaction or reduce maternal employment guilt.

Prospective and intervention studies could evaluate schedule predictability and access to childcare support, with parenting satisfaction and maternal employment guilt assessed as distinct outcomes. Research could also examine parental leave and childcare provision in relation to employees’ parenting experiences, well-being, and retention. No workplace intervention or policy effect was evaluated in the present study; these are areas for further investigation rather than recommendations whose effectiveness is established by the current findings.

## 8. Conclusions

Among nurses and midwives with young children, maternal employment guilt was inversely associated with parenting satisfaction. Supplementary exploratory comparisons showed that the satisfaction–MEGS correlation was significantly more negative than the corresponding correlations for efficacy and interest after Holm correction. However, these findings do not establish a unique or causal role for satisfaction. The association between total PSOC and MEGS was not statistically significant in the covariate-adjusted linear model, whose overall test was also nonsignificant. Exploratory diagnostics suggested possible nonlinearity, requiring confirmation.

Differences in PSOC scores by work schedule and weekly working hours were exploratory and should be regarded as hypothesis-generating. These findings support further investigation of parenting satisfaction and work–family conditions, but do not establish that changing work arrangements would improve parenting competence or reduce maternal employment guilt.

## Figures and Tables

**Table 1:** Sociodemographic and work characteristics of participants.

Characteristics (N = 150)	*n*	%
**Age (years)** ≤30 ≥31	64 86	42.7 57.3
**Education** High school University	19 131	12.7 87.3
**Marital Status** Married Single	146 4	97.3 2.7
**Family type** Nuclear Extended	137 13	91.3 8.7
**Income level** Good Middle Poor	71 75 4	47.3 50.0 2.7
**Number of children** 1 2 3	74 64 12	49.3 42.7 8.0
**Age of youngest child (years)** 1–2 3–4 5–6	31 66 53	20.7 44.0 35.3
**Personal health problem** Yes No	33 117	22.0 78.0
**Presence of a health problem in the child** Yes No	14 136	9.3 90.7
**Childcare support** Yes No	132 18	88.0 12.0
**Work experience (years)** 0–5 6–10 11–14 ≥15	13 66 43 28	8.7 44.0 28.7 18.7
**Work unit** Ambulatory Operating room/emergency Inpatient Intensive care	30 20 46 54	20.0 13.3 30.7 36.0
**Work schedule** Only during daytime Shift	45 105	30.0 70.0
**Weekly working hours** 40 ≥45	63 87	42.0 58.0
**Unpaid maternity leave** Yes No	107 43	71.3 28.7
**Unpaid maternity leave duration (years)** 1 2	68 36	65.4 34.6

Note. Percentages for unpaid maternity leave duration are based on 104 respondents; duration was missing for three of the 107 participants who reported taking unpaid leave.

**Table 2:** Descriptive statistics for PSOC and MEGS scores (N = 150).

Scale/Subscale	Mean ± SD	Minimum	Maximum
PSOC efficacy	27.68 ± 3.87	12	35
PSOC interest	6.85 ± 2.43	2	10
PSOC satisfaction	21.36 ± 5.53	9	34
PSOC total	55.89 ± 7.47	41	74
MEGS total	62.27 ± 15.72	15	90

Note. PSOC: Parenting Sense of Competence Scale–Mother Form; MEGS: Maternal Employment Guilt Scale; SD: standard deviation. Minimum and maximum are observed sample scores.

**Table 3:** Comparison of PSOC and MEGS scores by sociodemographic and work-related characteristics.

Characteristic/Group	PSOC Efficacy		PSOC Interest		PSOC Satisfaction		PSOC Total		MEGS Total	
	Mean ± SD	Statistic *p*	Mean ± SD	Statistic *p*	Mean ± SD	Statistic *p*	Mean ± SD	Statistic *p*	Mean ± SD	Statistic *p*
**Age**										
(1) ≤30 (*n* = 64)	28.06 ± 2.89	U = 2861.0 *p* = 0.678	6.03 ± 2.73	U = 1957.5 ***p*** **= 0.002**	19.59 ± 5.72	t = −3.497 ***p*** **< 0.001**	53.69 ± 7.33	t = −3.207 ***p*** **= 0.002**	64.69 ± 13.23	t = 1.631 *p* = 0.105
(2) ≥31 (*n* = 86)	27.40 ± 4.46		7.45 ± 2.00		22.67 ± 5.03		57.52 ± 7.18		60.48 ± 17.21	
**Education**										
(1) High school (*n* = 19)	28.68 ± 2.96	Z = 0.773 *p* = 0.441	4.95 ± 2.50	Z = −3.343 ***p*** **< 0.001**	16.11 ± 5.67	t = −4.737 ***p*** **< 0.001**	49.74 ± 6.51	t = −4.034 ***p*** **< 0.001**	66.26 ± 7.54	t = 2.028 ^a^ ***p*** **= 0.048**
(2) University (*n* = 131)	27.53 ± 3.97		7.12 ± 2.30		22.12 ± 5.10		56.78 ± 7.19		61.69 ± 16.52	
**Family type**										
(1) Nuclear (*n* = 137)	27.61 ± 3.95	Z = −0.020 *p* = 0.987	6.86 ± 2.45	Z = 0.253 *p* = 0.803	21.50 ± 5.54	t = 1.032 *p* = 0.304	55.98 ± 7.45	t = 0.486 *p* = 0.628	61.65 ± 15.94	t = −1.585 *p* = 0.115
(2) Extended (*n* = 13)	28.38 ± 2.84		6.69 ± 2.36		19.85 ± 5.44		54.92 ± 7.87		68.85 ± 11.79	
**Income level**										
(1) Good (*n* = 71)	28.17 ± 3.18	H = 2.819 *p* = 0.244	6.69 ± 2.68	H = 0.468 *p* = 0.791	20.32 ± 5.85	F = 4.490 ***p*** **= 0.013** [1, 2]	55.18 ± 7.82	F = 3.114 ***p*** **= 0.047**	64.34 ± 12.82	F = 4.144 ^a^ *p* = 0.052
(2) Middle (*n* = 75)	27.35 ± 4.37		7.03 ± 2.19		22.57 ± 5.00		56.95 ± 6.70		60.87 ± 18.11	
(3) Poor (*n* = 4)	25.25 ± 4.50		6.25 ± 2.50		17.00 ± 4.00		48.50 ± 11.00		52.00 ± 8.00	
**Number of children**										
(1) 1 (*n* = 74)	27.45 ± 3.81	H = 5.646 *p* = 0.059	6.81 ± 2.46	H = 0.040 *p* = 0.980	21.11 ± 5.76	F = 0.287 *p* = 0.751	55.36 ± 7.49	F = 0.414 *p* = 0.662	63.22 ± 16.75	F = 0.304 ^a^ *p* = 0.740
(2) 2 (*n* = 64)	27.62 ± 3.97		6.89 ± 2.41		21.75 ± 5.32		56.27 ± 7.39		61.83 ± 13.32	
(3) 3 (*n* = 12)	29.42 ± 3.53		6.83 ± 2.55		20.83 ± 5.59		57.08 ± 8.15		58.83 ± 21.22	
**Age of youngest child**										
(1) 1–2 (*n* = 31)	28.29 ± 3.04	H = 0.482 *p* = 0.786	6.87 ± 2.51	H = 1.819 *p* = 0.403	22.68 ± 5.51	F = 1.138 *p* = 0.323	57.84 ± 8.07	F = 1.341 *p* = 0.265	64.61 ± 17.74	F = 0.434 *p* = 0.649
(2) 3–4 (*n* = 66)	27.89 ± 3.06		6.58 ± 2.46		20.91 ± 5.45		55.38 ± 7.24		61.79 ± 12.82	
(3) 5–6 (*n* = 53)	27.06 ± 5.02		7.17 ± 2.35		21.15 ± 5.64		55.38 ± 7.34		61.51 ± 17.82	
**Health problem**										
(1) Yes (*n* = 33)	27.45 ± 4.01	Z = −0.126 *p* = 0.902	6.00 ± 2.68	Z = −2.059 ***p*** **= 0.040**	19.52 ± 6.08	t = −2.196 ***p*** **= 0.030**	52.97 ± 7.91	t = −2.589 ***p*** **= 0.011**	61.61 ± 15.34	t = −0.275 *p* = 0.784
(2) No (*n* = 117)	27.74 ± 3.84		7.09 ± 2.31		21.88 ± 5.28		56.71 ± 7.16		62.46 ± 15.89	
**Child health problem**										
(1) Yes (*n* = 14)	24.07 ± 6.22	Z = −2.239 ***p*** **= 0.025**	7.29 ± 0.99	Z = 0.010 *p* = 0.995	24.29 ± 3.10	t = 3.363 ^a^ ***p*** **= 0.003**	55.64 ± 4.85	t = −0.185 ^a^ *p* = 0.855	50.57 ± 18.19	t = −3.002 ***p*** **= 0.003**
(2) No (*n* = 136)	28.05 ± 3.36		6.80 ± 2.53		21.06 ± 5.65		55.91 ± 7.70		63.48 ± 15.01	
**Childcare support**										
(1) Yes (*n* = 132)	27.72 ± 3.69	Z = −0.259 *p* = 0.798	6.80 ± 2.49	Z = −0.391 *p* = 0.698	20.98 ± 5.68	t = −3.376 ^a^ ***p*** **= 0.002**	55.50 ± 7.64	t = −1.729 *p* = 0.086	62.89 ± 15.03	t = 1.296 *p* = 0.197
(2) No (*n* = 18)	27.39 ± 5.12		7.22 ± 2.02		24.11 ± 3.32		58.72 ± 5.37		57.78 ± 20.06	
**Work experience**										
(1) 0–5 (*n* = 13)	27.92 ± 2.60	H = 2.020 *p* = 0.568	6.38 ± 3.01	H = 4.414 *p* = 0.220	20.31 ± 5.98	F = 4.306 *p* = 0.006 [2–4, 3–4]	54.62 ± 8.79	F = 2.579 *p* = 0.056	67.85 ± 7.90	F = 2.712 ^a^ *p* = 0.054
(2) 6–10 (*n* = 66)	28.24 ± 3.23		6.42 ± 2.52		20.45 ± 5.32		55.12 ± 7.25		62.41 ± 16.12	
(3) 11–14 (*n* = 43)	27.07 ± 4.25		7.16 ± 2.27		20.95 ± 6.07		55.19 ± 7.78		64.28 ± 13.70	
(4) ≥15 (*n* = 28)	27.18 ± 4.97		7.57 ± 2.01		24.61 ± 3.74		59.36 ± 6.09		56.29 ± 18.98	
**Work unit**										
(1) Ambulatory (*n* = 30)	27.27 ± 4.30	H = 2.099 *p* = 0.552	8.20 ± 1.42	H = 13.968 ***p*** **= 0.003** [1–3, 1–4]	24.10 ± 4.17	F = 4.946 ***p*** **= 0.003** [1–4]	59.57 ± 7.44	F = 5.022 ***p*** **= 0.002** [1–4]	61.67 ± 13.48	F = 0.490 *p* = 0.690
(2) Operating room/emergency (*n* = 20)	28.55 ± 2.86		7.25 ± 2.34		22.00 ± 3.84		57.80 ± 5.33		65.35 ± 12.30	
(3) Inpatient (*n* = 46)	27.24 ± 4.09		6.63 ± 2.43		21.48 ± 5.78		55.35 ± 7.04		60.52 ± 17.24	
(4) Intensive care (*n* = 54)	27.96 ± 3.77		6.13 ± 2.62		19.50 ± 5.91		53.59 ± 7.71		62.96 ± 16.80	
**Work schedule**										
(1) Day only (*n* = 45)	27.58 ± 4.06	Z = −0.640 *p* = 0.524	7.78 ± 1.80	Z = 2.804 ***p*** **= 0.005**	23.58 ± 4.52	t = 3.320 ***p*** **= 0.001**	58.93 ± 7.33	t = 3.384 ***p*** **< 0.001**	62.36 ± 16.34	t = 0.042 *p* = 0.967
(2) Shift (*n* = 105)	27.72 ± 3.80		6.45 ± 2.56		20.41 ± 5.68		54.58 ± 7.17		62.24 ± 15.53	
**Weekly working hours**										
(1) 40 (*n* = 63)	27.21 ± 4.12	Z = −1.596 *p* = 0.111	7.63 ± 1.91	Z = 3.003 ***p*** **= 0.003**	22.92 ± 4.54	t = 3.147 ^a^ ***p*** **= 0.002**	57.76 ± 6.92	t = 2.671 ***p*** **= 0.008**	61.67 ± 18.54	t = −0.381 ^a^ *p* = 0.704
(2) ≥45 (*n* = 87)	28.02 ± 3.66		6.28 ± 2.61		20.23 ± 5.93		54.53 ± 7.59		62.71 ± 13.42	
**Unpaid maternity leave**										
(1) Yes (*n* = 107)	27.92 ± 3.85	Z = 1.587 *p* = 0.113	6.61 ± 2.51	Z = −1.775 *p* = 0.076	20.65 ± 5.89	t = −2.924 ^a^ ***p*** **= 0.004**	55.18 ± 7.66	t = −1.850 *p* = 0.066	62.25 ± 14.58	t = −0.026 *p* = 0.980
(2) No (*n* = 43)	27.09 ± 3.90		7.44 ± 2.13		23.12 ± 4.07		57.65 ± 6.74		62.33 ± 18.45	
**Unpaid maternity leave duration**										
(1) 1 year (*n* = 68)	27.62 ± 3.76	Z = −1.704 *p* = 0.089	6.66 ± 2.35	Z = 0.052 *p* = 0.962	21.04 ± 5.41	t = 0.957 *p* = 0.341	55.32 ± 7.32	t = 0.300 *p* = 0.765	63.57 ± 14.93	t = 1.180 *p* = 0.241
(2) 2 years (*n* = 36)	28.44 ± 4.04		6.53 ± 2.81		19.89 ± 6.62		54.86 ± 7.75		60.06 ± 13.52	

Note. Values are mean ± SD. U and Z denote Mann–Whitney statistics; t, F, and H denote independent-samples t, one-way ANOVA, and Kruskal–Wallis statistics. Superscript a indicates Welch’s test. Brackets identify significant post hoc pairs using the numbered categories within each characteristic. Adjusted pairwise *p* values are in Table S4. Overall *p* values are nominal; Tukey–Kramer and Dunn–Bonferroni adjustments apply within each post hoc family only. Bivariate findings are exploratory.

**Table 4:** Correlation between PSOC and MEGS.

PSOC Score	ρ	95% CI	*p*
Efficacy	0.065	−0.112 to 0.239	0.431
Interest	−0.063	−0.221 to 0.095	0.447
Satisfaction	−0.324	−0.466 to −0.162	<0.001
Total	−0.177	−0.336 to −0.013	0.030

Note. Spearman’s rank correlation; confidence intervals were estimated using 10,000 bootstrap resamples.

**Table 5:** Multivariable linear regression of factors associated with maternal employment guilt.

Predictor	B	Robust SE	β	95% CI	z	*p*
Constant	91.252	15.888		60.112 to 122.392	5.743	<0.001
PSOC total	−0.456	0.252	−0.216	−0.950 to 0.038	−1.808	0.071
Age ≥ 31 years	−1.558	2.827	−0.049	−7.098 to 3.983	−0.551	0.582
University education	−0.116	2.863	−0.002	−5.728 to 5.495	−0.041	0.968
Child health problem present	−12.767	5.376	−0.237	−23.304 to −2.230	−2.375	0.018
Childcare support present	1.754	5.218	0.036	−8.472 to 11.980	0.336	0.737
Shift work	−2.999	5.359	−0.088	−13.502 to 7.505	−0.560	0.576
Weekly hours ≥ 45	1.517	4.399	0.048	−7.105 to 10.140	0.345	0.730
Unpaid maternity leave used	−1.634	3.448	−0.047	−8.392 to 5.125	−0.474	0.636
Operating room/emergency	3.574	4.785	0.078	−5.805 to 12.954	0.747	0.455
Inpatient unit	−2.734	4.701	−0.080	−11.947 to 6.479	−0.582	0.561
Intensive care unit	−0.343	5.164	−0.011	−10.465 to 9.779	−0.066	0.947

Note. Dependent variable: MEGS total score. Main independent variable: total PSOC score, entered continuously. Binary covariates were coded 0 for age ≤ 30 years, high-school education, no child health problem, no childcare support, day-only work, 40 weekly hours, and no unpaid maternity leave; comparison categories were coded 1. Work unit used three dummy variables with ambulatory units as the reference. HC3-robust standard errors are reported. Coefficient *p* values use two-sided z tests with the standard normal reference distribution; 95% confidence intervals are B ± 1.96 × SE(HC3). Effective model N = 150 (complete cases); 11 predictor coefficients, excluding the intercept; R^2^ = 0.124; adjusted R^2^ = 0.054; HC3-robust Wald F(11,138) = 1.659, *p* = 0.089. All variance inflation factors were <3.

## Data Availability

The datasets used and analyzed during this study are not publicly available because combinations of demographic, work-related, and health information could compromise participant confidentiality, particularly within small subgroups. De-identified data are available from the corresponding author upon reasonable request, subject to appropriate safeguards for participant confidentiality.

## Supplementary Materials

The following supporting information can be downloaded at: https://www.mdpi.com/article/10.3390/healthcare14213568/s1, Table S1. Exploratory associations between PSOC dimensions and maternal employment guilt after covariate adjustment (N = 150). Table S2. Exploratory comparisons of Spearman correlations with maternal employment guilt. Table S3. Diagnostics and exploratory sensitivity analyses for the adjusted PSOC–MEGS model. Figure S1. Diagnostic plots for the adjusted linear model. Table S4. Adjusted post hoc pairwise *p* values for Table 3.
