# Supplementary material for: Parenting Sense of Competence and Maternal Employment Guilt Among Female Healthcare Professionals with Young Children in Türkiye: A Cross-Sectional Study
Source: Healthcare (Basel). 2026 Oct 22;14(21):3568. doi: 10.3390/healthcare14213568 (PMC13762838; doi:10.3390/healthcare14213568)
Supplement: Supplementary file 1 [file healthcare-14-03568-s001.zip › Supplementary_Table_S1.pdf]

Supplementary Table S1

Exploratory associations between PSOC dimensions and maternal employment guilt after covariate adjustment (N=150).

PSOC dimension	B	HC3 SE	95% CI	p	Holm-adjusted p
Efficacy	0.130	0.577	−1.000 to 1.260	0.822	1.000
Interest	−0.239	0.587	−1.389 to 0.912	0.685	1.000
Satisfaction	−0.916	0.326	−1.555 to −0.277	**0.005**	**0.015**

Note. Each dimension was entered into a separate linear regression with MEGS as the dependent variable, adjusting for age, education, child health status, childcare support, work schedule, weekly working hours, unpaid maternity leave use, and work unit. Age was grouped as ≤30/≥31 years and weekly working hours as 40/≥45 hours. Each model included 11 predictor coefficients, excluding the intercept. B denotes an unstandardized coefficient. Confidence intervals and coefficient p values use HC3-robust standard errors and a normal approximation. Holm adjustment applies to the three dimension-specific coefficient tests. These supplementary analyses were undertaken during revision and were not prespecified.

Overall HC3-robust model tests: efficacy, $F(11,138)=1.070$, $p=0.390$, $R^2=0.091$; interest, $F(11,138)=1.138$, $p=0.337$, $R^2=0.091$; satisfaction, $F(11,138)=1.694$, $p=0.081$, $R^2=0.161$. None of the overall model tests was statistically significant.

Abbreviations: PSOC, Parenting Sense of Competence Scale; MEGS, Maternal Employment Guilt Scale; SE, standard error; CI, confidence interval.

Covariate coding. Reference categories (0) were age ≤30 years, high-school education, no child health problem, no childcare support, day-only work, 40 weekly hours, and no unpaid maternity leave. Corresponding comparison categories were coded 1. Work unit was represented by three dummy variables, with ambulatory units as the reference. All covariates were entered together in each model.
