# Supplementary material for: Parenting Sense of Competence and Maternal Employment Guilt Among Female Healthcare Professionals with Young Children in Türkiye: A Cross-Sectional Study
Source: Healthcare (Basel). 2026 Oct 22;14(21):3568. doi: 10.3390/healthcare14213568 (PMC13762838; doi:10.3390/healthcare14213568)
Supplement: Supplementary file 1 [file healthcare-14-03568-s001.zip › Supplementary_Table_S2.pdf]

Table S2. Exploratory comparisons of Spearman correlations with maternal employment guilt

N=150; 50,000 paired bootstrap resamples.

Comparator	ρ Satisfaction	ρ Comparator	Difference Δρ	95% CI for Δρ	Bootstrap p	Holm-adjusted p
Efficacy	−0.324	0.065	−0.389	−0.618 to −0.153	0.00082	0.00246
Interest	−0.324	−0.063	−0.262	−0.415 to −0.112	0.00112	0.00246
Total PSOC	−0.324	−0.177	−0.147	−0.258 to −0.040	0.00868	0.00868

Note. PSOC, Parenting Sense of Competence Scale; MEGS, Maternal Employment Guilt Scale; CI, confidence interval. Each ρ is a correlation with MEGS. $\Delta\rho = \rho(\text{MEGS, satisfaction}) - \rho(\text{MEGS, comparator})$; negative values indicate a more negative correlation for satisfaction. Participants were resampled jointly with replacement, and ranks were recalculated in each resample using average ranks for ties (seed 20261017). CIs are unadjusted percentile bootstrap intervals. Approximate two-sided p values were calculated from the centered bootstrap distribution: $p = [1 + \text{count}(|\Delta\rho^* - \Delta\rho| \geq |\Delta\rho|)]/50{,}001$, where $\Delta\rho^*$ denotes a resampled difference. Holm correction was applied across the three comparisons in this table only. These tests compare signed coefficients, not their absolute magnitudes. The total PSOC score includes satisfaction items; its comparison is therefore a secondary part–whole comparison. Analyses were conducted following peer review and are exploratory. Satisfaction was selected for comparison after inspection of the observed correlations; Holm adjustment does not account for this selection.
