# Supplementary material for: Parenting Sense of Competence and Maternal Employment Guilt Among Female Healthcare Professionals with Young Children in Türkiye: A Cross-Sectional Study
Source: Healthcare (Basel). 2026 Oct 22;14(21):3568. doi: 10.3390/healthcare14213568 (PMC13762838; doi:10.3390/healthcare14213568)
Supplement: Supplementary file 1 [file healthcare-14-03568-s001.zip › Supplementary_Table_S3 and Figure S1_Diagnostics.pdf]

Supplementary regression diagnostics

Table S3. Diagnostics and exploratory sensitivity analyses for the adjusted PSOC–MEGS model.

Outcome: MEGS. Main predictor: total PSOC. Covariates and coding match Table 5. The original model includes 150 participants and 11 predictor coefficients, excluding the intercept. Diagnostics and sensitivity analyses were conducted during revision.

Assessment	Result
**Original linear model**	PSOC B=−0.456; 95% CI −0.950 to 0.038; p=0.071. HC3 Wald F(11,138)=1.659; p=0.089; R^2=0.124.
**Residual distribution**	Shapiro–Wilk W=0.986; p=0.146. Q–Q plot shows tail deviations; a nonsignificant test does not prove normality.
**Heteroskedasticity**	Koenker LM $\chi^2(11)$=25.826; p=0.007. HC3 inference retained.
**Externally studentized residuals**	Range −3.024 to 2.180; 1 observation with |residual|>3.
**Leverage**	Maximum 0.177; 1 observation >2k/n=0.160 (k=12, including intercept).
**Cook's distance**	Maximum 0.069; 12 observations >4/n=0.0267.
**Combined screening**	13 observations met at least one criterion. Screening did not determine main-analysis exclusions.

All coefficient intervals and p values below use HC3 standard errors with a normal reference distribution. Quadratic coefficients refer to $(\text{PSOC}-\text{mean PSOC})^2$; the PSOC mean of the full sample was retained for refits.

Analysis	N	Coefficient B (95% CI)	p
**Linear model, all observations**	150	−0.456 (−0.950 to 0.038)	0.071
**Linear model, flagged observations excluded**	137	−0.347 (−0.758 to 0.064)	0.098
**Linear model, leave-one-out refits**	149 each	B range: −0.543 to −0.358	0.026–0.135
**Added quadratic term, all observations**	150	−0.063 (−0.101 to −0.025)	0.001
**Quadratic term, flagged observations excluded**	137	−0.049 (−0.084 to −0.014)	0.007
**Quadratic term, leave-one-out refits**	149 each	B range: −0.068 to −0.054	0.0004–0.0064

The quadratic model retained the same covariates and added one coefficient: HC3 Wald F(12,137)=2.331, p=0.010; R^2=0.195. These post hoc analyses are exploratory and unadjusted for multiplicity. They do not validate a nonlinear mechanism or identify a clinical threshold. Main analyses retain all 150 observations.

Figure S1. Diagnostic plots for the adjusted linear model.

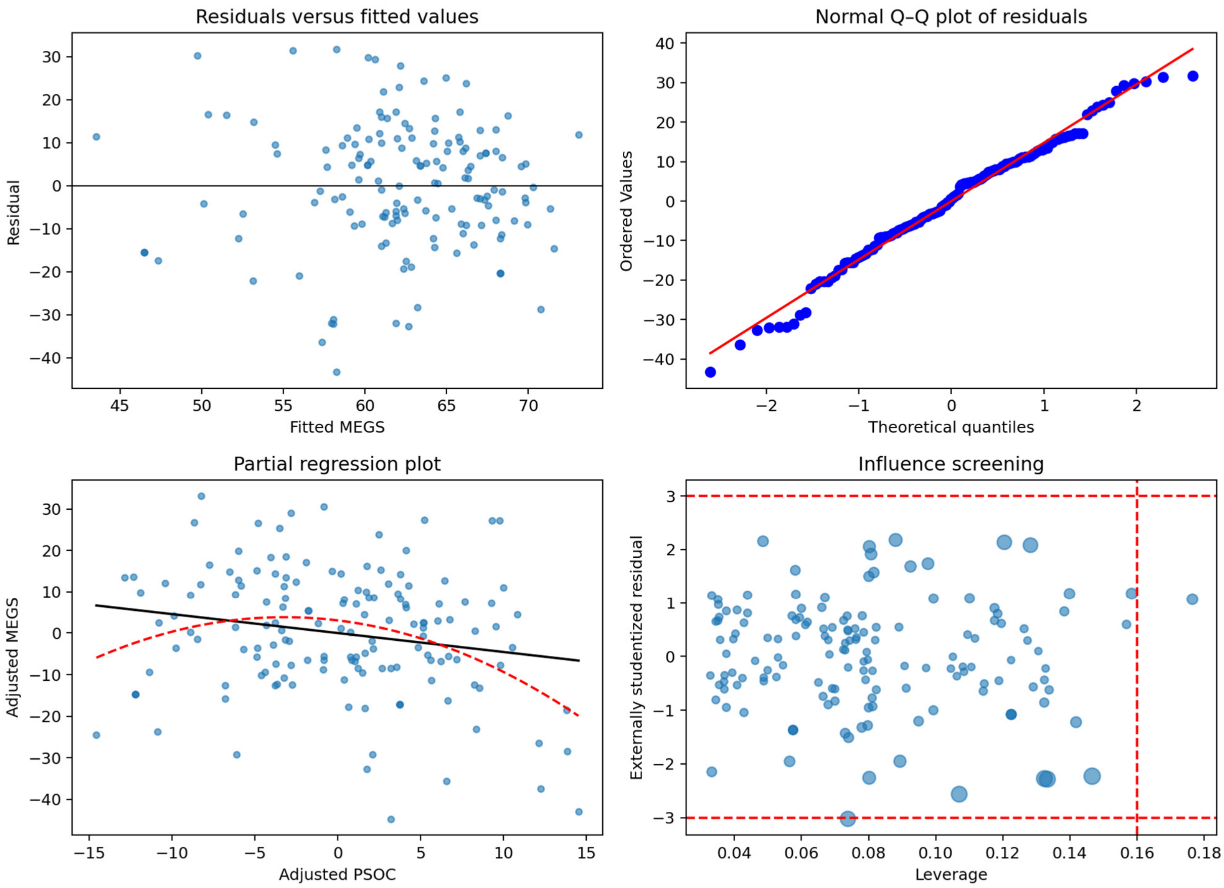

Upper left: residuals versus fitted MEGS. Upper right: normal Q–Q plot. Lower left: partial-regression plot adjusting PSOC and MEGS for the other covariates; the black line is linear and the red curve is a descriptive quadratic smoother. The formal quadratic test in Table S3 adds centered PSOC squared to the full covariate model. Lower right: leverage versus externally studentized residuals; marker area increases with Cook's distance. Dashed lines indicate screening thresholds, not automatic deletion rules.
