# Supplementary material for: Parenting Sense of Competence and Maternal Employment Guilt Among Female Healthcare Professionals with Young Children in Türkiye: A Cross-Sectional Study
Source: Healthcare (Basel). 2026 Oct 22;14(21):3568. doi: 10.3390/healthcare14213568 (PMC13762838; doi:10.3390/healthcare14213568)
Supplement: Supplementary file 1 [file healthcare-14-03568-s001.zip › Supplementary_Table_S4_Posthoc.pdf]

Supplementary pairwise comparisons

Table S4. Adjusted post hoc pairwise p values for Table 3.

All pairs are shown for the six score-by-characteristic comparisons with significant omnibus tests. Numbered groups match Table 3. Tukey–Kramer adjustment accounts for unequal group sizes; Dunn comparisons use pooled ranks with tie correction and Bonferroni adjustment across all six pairs for work unit.

Characteristic	PSOC score	Groups	Adjustment	Adjusted p
Income level	satisfaction	[1–2]	Tukey–Kramer	**0.035**
Income level	satisfaction	[1–3]	Tukey–Kramer	0.457
Income level	satisfaction	[2–3]	Tukey–Kramer	0.114
Income level	total	[1–2]	Tukey–Kramer	0.320
Income level	total	[1–3]	Tukey–Kramer	0.185
Income level	total	[2–3]	Tukey–Kramer	0.069
Work experience	satisfaction	[1–2]	Tukey–Kramer	1.000
Work experience	satisfaction	[1–3]	Tukey–Kramer	0.981
Work experience	satisfaction	[1–4]	Tukey–Kramer	0.083
Work experience	satisfaction	[2–3]	Tukey–Kramer	0.964
Work experience	satisfaction	[2–4]	Tukey–Kramer	**0.004**
Work experience	satisfaction	[3–4]	Tukey–Kramer	**0.029**
Work unit	satisfaction	[1–2]	Tukey–Kramer	0.523
Work unit	satisfaction	[1–3]	Tukey–Kramer	0.159
Work unit	satisfaction	[1–4]	Tukey–Kramer	**0.001**
Work unit	satisfaction	[2–3]	Tukey–Kramer	0.983
Work unit	satisfaction	[2–4]	Tukey–Kramer	0.281
Work unit	satisfaction	[3–4]	Tukey–Kramer	0.254
Work unit	total	[1–2]	Tukey–Kramer	0.829
Work unit	total	[1–3]	Tukey–Kramer	0.064
Work unit	total	[1–4]	Tukey–Kramer	**0.002**
Work unit	total	[2–3]	Tukey–Kramer	0.580
Work unit	total	[2–4]	Tukey–Kramer	0.118
Work unit	total	[3–4]	Tukey–Kramer	0.616
Work unit	interest	[1–2]	Dunn–Bonferroni	1.000
Work unit	interest	[1–3]	Dunn–Bonferroni	**0.041**
Work unit	interest	[1–4]	Dunn–Bonferroni	**0.002**
Work unit	interest	[2–3]	Dunn–Bonferroni	1.000
Work unit	interest	[2–4]	Dunn–Bonferroni	0.564
Work unit	interest	[3–4]	Dunn–Bonferroni	1.000

Groups: income, (1) Good, (2) Middle, (3) Poor; work experience, (1) 0–5, (2) 6–10, (3) 11–14, (4) ≥15 years; work unit, (1) ambulatory, (2) operating room/emergency, (3) inpatient, (4) intensive care. Bold indicates adjusted $p<0.05$. Adjustments apply within each post hoc family, not across all Table 3 tests. The significant income–total PSOC omnibus test had no pair reaching adjusted $p<0.05$. All comparisons remain exploratory.
